# Different moxibustion therapies for urinary retention after anorectal surgery: A protocol for systematic review and network meta-analysis

**DOI:** 10.1097/MD.0000000000024132

**Published:** 2021-01-15

**Authors:** Jinwen Hu, Yuepeng Sun, Lili Cao, Shulan Shen, Xiaoyang Hu

**Affiliations:** a Jiangxi University of Traditional Chinese Medicine; b Fuxin Traditional Chinese Medicine Hospital, Fuxin, Liaoning Province; c Affiliated Hospital of Jiangxi University of Traditional Chinese Medicine, Nanchang, Jiangxi Province, China.

**Keywords:** anorectal, moxibustion, network meta-analysis, postoperative urinary retention, protocol

## Abstract

**Background:**

Postoperative urinary retention is a disease that seriously affects human daily work and life, and greatly reduces people's quality of life and affects human health all over the world. Now, many studies have shown that moxibustion has a significant effect on postoperative urinary retention. In this study, network meta-analysis was used to analyze and compare the clinical efficacy and difference of different moxibustion treatments on postoperative urinary retention.

**Methods:**

Only randomized controlled trials (RCTs) will be included and all patients were diagnosed as postoperative urinary retention. Computer search Chinese databases: CNKI, Wanfang (WANFANG), VIP (VIP), Chinese Biomedical Literature Database (SinoMed), English database search PubMed, Cochrane library, Web of Science. The search period limit is from the time the date of database establishment to November 17, 2020. To avoid omissions, we will manually search for relevant reference materials and conference papers. The risk of bias in the final included studies will be assessed according to the guidelines of the Cochrane System Intervention Review Manual. All data analysis will be conducted by Revman5.3, Gemtc 0.14.3, and Stata 14.2.

**Results:**

The effectiveness of each intervention was quantified. The main results included effective rate, first urination time, and residual urine volume.

**Conclusion:**

Objective to provide evidence-based medicine basis for clinicians to choose more effective moxibustion therapy for postoperative urinary retention.

## 1 Introduction

Postoperative urinary retention (POUR) is one of the more common complications after surgery. It is also one of the common clinical emergencies at present. If it is not handled properly in time, it will not only bring great inconvenience to the life of the patient, but also affect the healing of the postoperative wound and prolong the hospitalization time. It may even cause serious consequences such as bladder rupture and kidney failure to endanger the patient Life.^[1]^ This disease is mainly manifested as the patient's bladder is filled with urine and cannot be discharged spontaneously, and the lower abdomen bladder area has full discomfort or pain, mostly caused by the development of dysuria to a certain extent.^[2]^ Studies have shown that the incidence of urinary retention after surgery is 2.1% to 3.8%, and it has been increasing year by year in recent years.^[3,4]^ In anorectal surgery, the incidence of urinary retention can be as high as 52%, and men are more likely to occur than women.^[5]^ The current clinical understanding of its pathogenesis is mainly summarized as: the influence of anesthesia, pain factors, excessive local fillers, psychological factors, excessive fluid replacement, postoperative analgesic pump use, etc.^[6]^ The most effective measure for current treatment of postoperative urinary retention is to retain catheterization, but it not only brings a psychological burden to the patient, but also increases the possibility of urinary system infection, and even urinary retention after removal of the catheter,^[7]^ so catheterization Not the most appropriate method to treat urinary retention after anorectal surgery. The application of anesthetics during surgery can block the genital and pelvic visceral nerves, which can easily lead to weak bladder muscle contraction and urethral sphincter spasm to cause abnormal urination.^[8]^ Therefore, intramuscular injections of anticholinerase drugs such as neostigmine are often used in clinical practice. Exciting the bladder to hold back the urinary muscles and promote urinary excretion.^[9]^ But due to the wide distribution of cholinergic nerves, the low selectivity of neostigmine may cause severe cholinergic crisis.^[10]^ Therefore, this method is limited in clinical use, so it is particularly important to explore treatments with high efficiency and few side effects.

The prevention and treatment measures of Chinese medicine for urinary retention after anorectal surgery currently include acupuncture treatment, moxibustion treatment, acupoint injection, ear-point pressing, acupoint massage, sticking therapy, medicine ironing therapy, etc. Clinical practice shows that Chinese medicine treats anorectal diseases after surgery. Urinary retention has outstanding curative effect, complete theory, and various treatment methods. Various treatment methods can also be applied in combination. However, the advantages of each treatment method are different, which brings confusion to the choice of clinical operators. In recent years, a number of clinical studies have shown that acupuncture and moxibustion treatments have achieved good results in the treatment of urinary retention after anorectal surgery.^[11–13]^ But there is no relevant research to prove it. These treatment methods can bring greater clinical effects. Therefore, this study aims to use the method of network meta-analysis to treat the 3 methods of moxibustion (thermal moxibustion, ginger moxibustion, gentle moxibustion) in order to rank the effectiveness of urinary retention after anorectal surgery, and provide scientific evidence-based medicine basis for clinical selection.

## 2 Protocol registration

The protocol of the systematic review has been registered in the INPLASY website (registration number is INPLASY2020110124). Our protocol will follow the Cochrane Handbook for Systematic Reviews of Interventions and the Preferred Reporting Items for Systematic Reviews and Meta-Analysis Protocol (PRISMA-P) statement guidelines.

## 3 Methods

### 3.1 Inclusion criteria

#### 3.1.1 Study type

Based on different moxibustion treatment of POUR RCTs, the language is limited in Chinese and English. Literature exclusion criteria: non RCTs literature, such as case report, literature review, etc; besides 3 kinds of moxibustion, other moxibustion or acupuncture therapies were used; the experimental group and the control group contained other interference therapy; only one document with the most complete information was selected for the repeated detection and repeated publication; literature with incomplete data or unable to obtain data and full text; documents suspected of counterfeiting.

#### 3.1.2 Participants

The patients diagnosed as POUR meet the internationally recognized diagnostic criteria and have clear curative effect criteria. There are no restrictions on age, race, sex, and source of cases. However, the following patients will be excluded:

(1) Patients who can’t tolerate moxibustion treatment;
(2) Patients with severe organic diseases;
(3) Pregnant women;
(4) Patients with mental illness or inability to accurately describe symptoms due to unconsciousness.

#### 3.1.3 Interventions

The experimental group was treated with thermal moxibustion, ginger moxibustion, gentle moxibustion. The control group was treated with conventional nursing or intramuscular injection of neostigmine. Both the experimental group and the control group could cooperate with conventional medical treatment.

#### 3.1.4 Outcome indicators

The included outcome indicators included one or more of the following: effective rate: refer to “Surgery Complications” and “People's Republic of China Traditional Chinese Medicine Industry Standards·Standards for Diagnostic Efficacy of TCM Diseases,” including 4 levels of recovery, markedly effective, effective and ineffective, effective rate = [(Healed + markedly effective + effective)/total number of cases] × 100%; first urination time. Residual urine volume.

### 3.2 Data sources and search strategies

Computer search Chinese databases: CNKI, Wanfang (WANFANG), VIP (VIP), Chinese Biomedical Literature Database (SinoMed), English database search PubMed, Cochrane library, Web of Science. Search terms are: “postoperative,” “urinary retention,” “difficult urination,” “anorectal,” “hemorrhoids,” “mixed hemorrhoids,” “acupuncture,” “moxibustion,” “thermal moxibustion,” “ginger moxibustion,” “gentle moxibustion.” The search time is from the establishment of the database to November 17, 2020. The search term is a combination of subject terms and free terms, and relevant references are manually searched. The retrieval strategy is shown in Table 1.

**Table 1:** Search strategy used in PubMed database.

Number	Search items
1	postoperative
2	urinary retention
3	difficult urination
4	1 or 2–3
5	anorectal
6	hemorrhoids
7	mixed hemorrhoids
8	5 or 6–7
9	acupuncture
10	moxibustion
11	thermal moxibustion
12	ginger moxibustion
13	gentle moxibustion
14	9 or 10–13
15	4 and 8 and 14

### 3.3 Selection of studies and data extraction

According to the literature inclusion and exclusion criteria, 2 reviewers independently screened all relevant literature, cross-checked the screening results, and handed over to the chief Chinese physician for consultation and decision in case of disagreement. Establish a document information extraction table in Excel. The extracted information includes: title, author, publication time, number of cases, average age, sex, intervention measures, and outcome indicators.

### 3.4 Risk assessment of bias

Two reviewers independently assessed the bias risk of the articles included in this study according to the Cochrane evaluator bias risk assessment tool. It includes selection bias, implementation bias, measurement bias, follow-up bias, reporting bias, and other sources bias. The evaluation results were evaluated as “high risk,” “low risk,” and “unclear risk.”^[14]^

### 3.5 Statistical analysis

Revman 5.3 software (Cochrane Collaboration) was used for bias evaluation. For continuous variables (time to first urination), the results will be reported as mean difference (MD) and 95% confidence interval (CI); count data (effective rate) will be compared by odds ratio (OR) and 95% CI are calculated. In the heterogeneity test, if *I*^2^ < 50%, *P* > .10, there is no significant heterogeneity. We chose the fixed effect model to combine the effect quantity. If the combined data is *I*^2^ > 50%, *P* < .10, it indicates high heterogeneity. We choose the random effect model to combine the effect quantity.^[15]^

Using Gemtc 0.14.3 (Developed by van Valkenhoef G et al) and Stata14.2 (STATA Corp., College Station, TX, USA) for mesh meta-analysis.^[16,17]^ In Gemtc software, Bayesian mesh meta-analysis is realized by Markov Chain Monte Carlo (MCMC) method, through 4 chains. For simulation, the number of iterations is set to 50,000, and the step size is set to 10.^[18]^ At the same time, the potential scale reduction parameter (potential scale reduced factor, PSRF) is used to evaluate the convergence of the results. When the PSRF is close to 1, it indicates that the results have good convergence and the obtained results are highly reliable.^[19]^

### 3.6 Assessment of inconsistency

There are many interventions involved in this study. In the evidence network of each outcome index, the closed loop formed by the research with direct evidence and indirect evidence needs to be assessmented of inconsistency by Stata software. The closed loop formed by the research with direct evidence and indirect evidence needs to be tested for inconsistency. Judge whether there is a local inconsistency, if there is no obvious inconsistency, the consistency model is adopted, otherwise, the inconsistency model is adopted. For the results obtained from the consistency model analysis, the stability of the results can be checked through the inconsistency model. Make evidence network diagrams, correction-comparison funnel diagrams and ranking diagrams in Stata software. The larger the value of surface under the cumulative ranking curves (SUCRA) and the area under the SUCRA curve, the greater the possibility that the intervention will become the best intervention the bigger.^[20]^

### 3.7 Sensitivity analysis

The purpose of sensitivity analysis is to eliminate low-quality research and explore sources of heterogeneity. Then, analyze the reliability and stability of the results by observing the heterogeneity of different studies and whether the results have changed after treatment.

### 3.8 Assessment of publication bias

If outcome indicators are included in the study ≥10, funnel plot will be used to assess publication bias for inclusion in the trial.^[21]^ If there are differences in symmetry or distribution, there will be publication bias or small sample effect.

### 3.9 Ethics and dissemination

Because this is a systematic review of the protocol and a network meta-analysis, all the data in this study are from published studies and do not involve patients, so there is no need for ethical recognition. The results of this study will be distributed to peer reviews and presented at relevant meetings.

## 4 Discussion

At present, western medicine treatment of postoperative urinary retention mostly uses psychological counseling, induction methods, physical therapy equipment, and drug treatment. If the above treatment methods are ineffective, urinary catheterization is used. Although catheterization can reduce the pain of patients to a certain extent, repeated catheterization can bring infection risks. Moreover, compared with commonly used clinical urinary catheterization and oral medication, moxibustion therapy has the advantages of small side effects, low cost, and convenient operation. In recent years, it has gradually gained the general recognition of clinicians. The disadvantage is that with the continuous development and improvement of traditional Chinese medicine treatment, although there are a variety of moxibustion therapies that can be used to treat urinary retention after anorectal surgery, there are few direct comparison trials of related therapies. To this end, this study included the 3 most commonly used clinical methods to do comparative research by looking up the literature and screen out the best treatment methods, hoping to provide some references for related treatment work in the future. These results can provide the basis for clinicians to determine the treatment plan for patients with POUR.

## Author contributions

**Conceptualization:** Jinwen Hu, Xiaoyang Hu, Yuepeng Sun, Lili Cao.

**Data curation:** Jinwen Hu, Lili Cao, Shulan Shen, Xiaoyang Hu.

**Formal analysis:** Jinwen Hu, Yuepeng Sun.

**Project administration:** Jinwen Hu, Lili Cao.

**Supervision:** Xiaoyang Hu, Lili Cao.

**Writing – review & editing:** Jinwen Hu, Yuepeng Sun, Shulan Shen.

## Abbreviations

Abbreviations: POUR = postoperative urinary retention, RCTs = randomized controlled trials.
